# Photoelectron Velocity Map Imaging Spectroscopy of the Beryllium Trimer and Tetramer

**DOI:** 10.1021/acs.jpclett.3c02169

**Published:** 2023-09-12

**Authors:** Noah B. Jaffe, John F. Stanton, Michael C. Heaven

**Affiliations:** † Department of Chemistry, Emory University, Atlanta, Georgia 30322, United States; ‡ Department of Chemistry - Quantum Theory Project, University of Florida, Gainesville, Florida 32611, United States

## Abstract

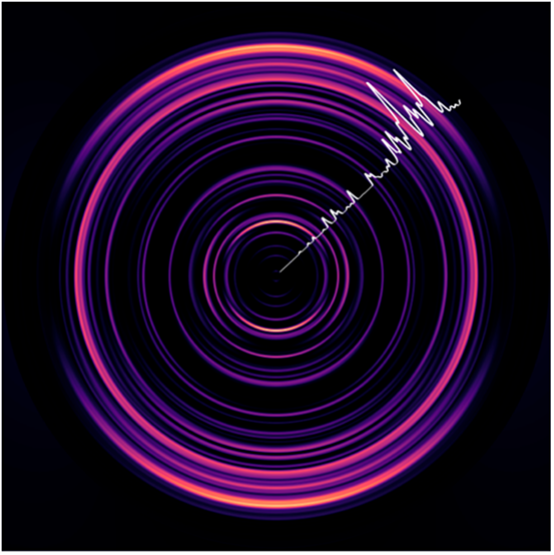

Computational studies of small beryllium clusters (Be_*N*_) predict dramatic, nonmonotonic changes in the bonding mechanisms and per-atom cohesion energies with increasing *N*. To date, experimental tests of these quantum chemistry models are lacking for all but the Be_2_ molecule. In the present study, we report spectroscopic data for Be_3_ and Be_4_ obtained via anion photodetachment spectroscopy. The trimer is predicted to have *D*_3*h*_ symmetric equilibrium structures for both the neutral molecule and the anion. Photodetachment spectra reveal transitions that originate from the X^2^A_2_″ ground state and the 1^2^A_1_′ electronically excited state. The state symmetries were assigned on the basis of anisotropic photoelectron angular distributions. The neutral and anionic forms of Be_4_ are predicted to be tetrahedral. Franck–Condon diagonal photodetachment was observed with a photoelectron angular distribution consistent with the expected Be_4_^–^X^2^A_1_ → Be_4_X^1^A_1_ transition. The electron affinities of Be_3_ and Be_4_ were determined to be 11363 ± 60 and 13052 ± 50 cm^–1^, respectively.

Small beryllium clusters have been the focus of many theoretical studies, with a particular focus on clusters that contain between two and six atoms.^1−23^ The bonding in this series is predicted to exhibit dramatic changes as the size of the cluster increases. Be_2_ and Be_3_ are found to be unbound at the Hartree–Fock self-consistent field (SCF) level of theory.^1,24−26^ Bound potential energy wells develop when sufficiently high-level treatments of electron correlation are applied.^1^ Be_4_ is the first member of the series to be bound at the SCF level. Experimental measurements^20,21,27^ show that Be_2_, with a formal bond order of 0, is bound with *D*_e_ = 934.9 cm^–1^. This value is appreciably greater than would be expected for a van der Waals interaction between two light closed-shell atoms (e.g., the binding energy for Ne_2_ is 29 cm^–1^ ^28^). The equilibrium bond length for Be_2_ of 2.444 Å is also indicative of a stronger than van der Waals interaction. High-level electronic structure calculations predict that the energy needed to remove an atom from the cluster (Be_*N*_ → Be_*N*–1_ + Be) has a nonmonotonic dependence on *N*. For example, Ascik et al.^8^ reported atom removal energies of 945, 8470, 22 400, and 13 830 cm^–1^ for *N* = 2, 3, 4, and 5. This trend is exhibited in the results from both *ab inito* models^7,8,29,30^ and density functional theory (DFT)^15,31,32^ calculations. A Jellium model^16,32−35^ that predicts a superatom closed-shell configuration for Be_4_ has been invoked to explain the high dissociation energy for Be_4_ and the relatively small dissociation energy for Be_5_.

Despite considerable interest in the bonding of Be_N_ clusters, Be_2_ is the only member of the series for which spectroscopic data have been reported.^20,21,27,36−38^ To date, our attempts to record electronic spectra for the *N* > 2 clusters using laser excitation techniques have been unsuccessful. However, Thomas et al.^39^ were able to obtain spectroscopic data for Mg_*N*_ clusters with *N* = 3–35 using electron photodetachment spectroscopy of size-selected Mg_*N*_^–^ cluster anions. For each cluster, they measured the electron affinity (EA) and observed an energy gap between the threshold detachment peak and the onset of features arising from electronically excited states of the neutral cluster. They interpreted the energy intervals as being approximate indicators of the HOMO–LUMO gaps in a Koopman’s approximation model. Thomas et al.^39^ equated the closing of the HOMO–LUMO gap with the emergence of metallic character.^39,40^ The feasibility of performing similar measurements for Be_N_ clusters was apparent as there were mass spectrometric observations of Be_*N*_^–^ clusters for the range *N* = 2–6.^41^ The smaller anions have also been the targets of several theoretical studies.^19,25,42,43^ Notably, Jordan and Simons^25,42^ used SCF models to examine the *N* = 2, 3, and 4 clusters. They found that the addition of correlation corrections was essential for Be_2_^–^, but they believed that SCF was sufficient to provide useful insights for the *N* = 3 and 4 clusters. It is worth noting that later studies performed with correlative treatment gave differing results, both qualitatively and quantitatively, especially with respect to the trimer and its anion.

More recent calculations have yielded EAs of 4030,^2^ 11 125,^43^ and 13 530^19^ cm^–1^ for *N* = 2, 3, and 4. Furthermore, the equilibrium structures of the *N* = 3 and 4 anions and their respective neutrals belong to the same point groups (*D*_3*h*_ for *N* = 3 and *T*_d_ for *N* = 4). Adding an electron to Be_3_ is predicted to shorten the bond length, while the bond length of Be_4_ is almost unaffected by hosting an extra electron.

Given this history, we used photodetachment spectroscopy of size-selected anions to examine the Be_3_ and Be_4_ clusters. In these experiments, we applied a slow electron velocity map imaging (SEVI) technique that reveals both the kinetic energies and spatial anisotropies of the photoelectrons.^44^ New electronic structure calculations have been carried out using equation of motion electron attachment (EOMEA) coupled cluster formalisms.^45,46^ For Be_3_^–^, these calculations were also used to examine the radiative properties of a long-lived electronically excited state.

The pulsed laser ablation technique used to generate Be_*N*_^–^ anions provided workable signal levels for Be_3_^–^ and notably more intense signals for Be_4_^–^. Be_2_^–^ was intermittently present in the ablation products, but the concentration was too low for reliable imaging measurements. Figure 1 shows a survey photoelectron spectrum of Be_3_^–^ with an inset velocity map image. Linearly polarized light was used for photodetachment, and the double-headed arrow next to the image shows the orientation of the polarization. The electron binding energy is given by the difference between the energy of the detachment photon and the kinetic energy of the photoelectron. Figure 1 is dominated by two prominent features, separated by approximately 2500 cm^–1^ in energy. Interestingly, with guidance from the high-level theory predictions shown in Table 1, we assign the lower binding energy feature to the Be_3_^–^1^2^A_1_′ → Be_3_X^1^A_1_′ transition. This assignment is further supported by the parallel anisotropy present in the velocity map image (anisotropy parameter^44^ β = 2), consistent with an A_1_′ → A_1_′ transition. We have not attempted to assign the weaker features of Figure 1 as they were not reproducible in successive measurements.

**Figure 1:**
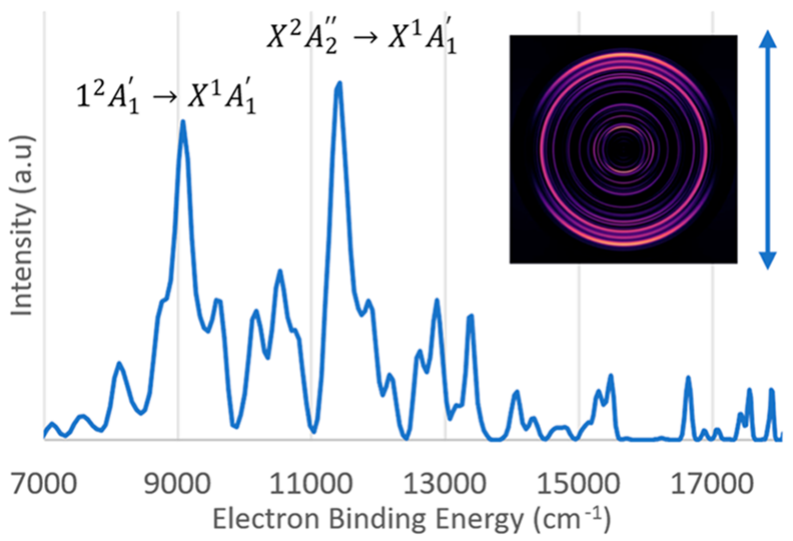
Photoelectron survey spectrum of the Be_3_^–^ anion taken with detachment photon energy of 18 797 cm^–1^ (vertical polarization). The strongest electronic transitions have been labeled with guidance from high-level electronic structure calculations. Inset photo shows the velocity map image produced by MEVELER software

**Table 1:** Calculated of Transition Energies^a^ for Be_3_^–^

transition	method	basis	energy (cm^–1^)	Δ*E*_exp_
Be_3_^–^X^2^A_2_″→Be_3_X^1^A_1_′	EOMEA-CCSD	aug-cc-pCVTZ	10193	–1170
	EOMEA-CCSD	aug-cc-pCVQZ	10 408	–955
	EOMEA-CCSDT	aug-cc-pCVTZ	11 575	212
	EOMEA-CCSDT	aug-cc-pCVQZ	11 774	411
	MRCI	aug-cc-pV5Z	11 125	–238
	experiment		11 363 ± 60	
Be_3_^–^1^2^A_1_′→Be_3_X^1^A_1_′	EOMEA-CCSD	aug-cc-pCVTZ	7912	–872
	EOMEA-CCSD	aug-cc-pCVQZ	8073	–711
	EOMEA-CCSDT	aug-cc-pCVTZ	8843	59
	EOMEA-CCSDT	aug-cc-pCVQZ	9001	217
	MRCI	aug-cc-pV5Z	8226	–558
	experiment		8784 ± 80	

a Comparison of computational and experimental energies. EOM-based methods were computed using the CFOUR package, while MRCI energies are taken from Kalemos.^43^ Computational results do not include zero point corrections. Δ*E*_exp_ lists the difference between the experimental and computational values for the computational results. Zero point corrections for EOMEA-CCSDT using an aug-cc-pCVTZ basis set are +77 cm^–1^ for the Be_3_^–^X^2^A_2_″ → Be_3_X^1^A_1_′ transition and +5 cm^–1^ for the Be_3_^–^1^2^A_1_′ → Be_3_X^1^A_1_′ transition.

An important characteristic of the SEVI technique is that the resolution improves as the photoelectron kinetic energy is reduced.^44^ A higher-resolution spectrum of the feature near 9000 cm^–1^ was obtained using 11 236 cm^–1^ (890 nm) detachment photons. The result, shown in Figure 2 along with an inset velocity map image, defined an electron binding energy of 8784 ± 40 cm^–1^. Multiple images recorded with the same conditions were analyzed, and the energies of the assignable features were averaged. The quoted uncertainties are 2σ errors that were determined by converting the uncertainties in pixel space from the raw image to the equivalent error in the electron kinetic energy.

**Figure 2:**
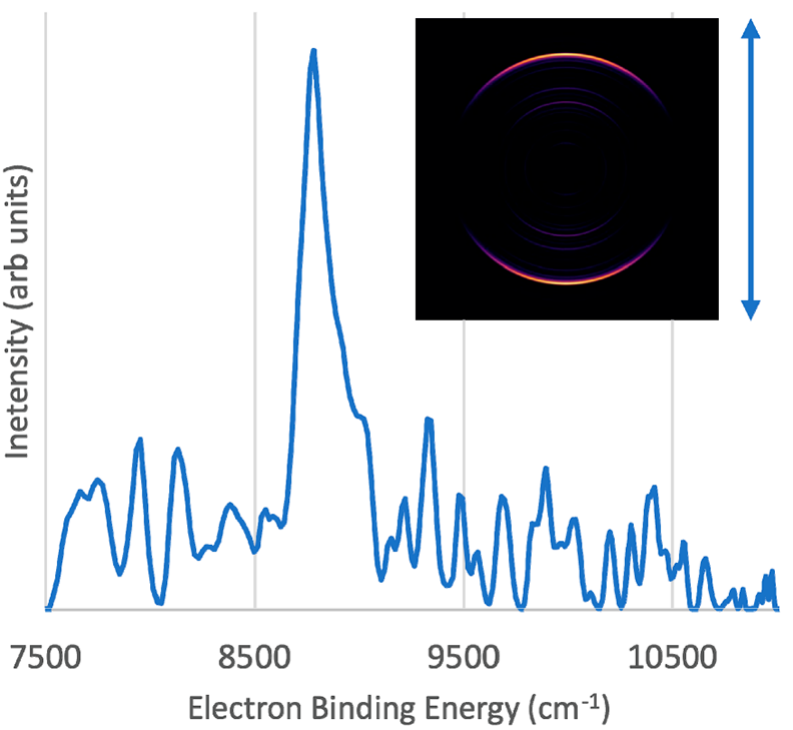
Photoelectron spectrum of Be_3_^–^ taken with a detachment photon energy of 11 236 cm^–1^ (vertical polarization). Inset photo shows the velocity map image produced by MEVELER software

The second prominent peak in Figure 1 is assigned to the Be_3_^–^X^2^A_2_″ → Be_3_X^1^A_1_′ origin transition. A higher-resolution spectrum and image for the Be_3_^–^X^2^A_2_″ → Be_3_X^1^A_1_′ transition is presented in Figure 3. This measurement yielded an EA of 11 363 ± 60 cm^–1^, in good agreement with the present EOMEA-CCSDT calculations (see below) and previously reported MRCI results.^43^ The image also shows a reasonable degree of perpendicular anisotropy, with β = −0.5 ± 0.3, which is supportive of the predicted symmetries of the electronic states involved in this transition. Interestingly, because smaller intensity peaks energetically just below the EA peak have near parallel anisotropy, the anisotropy of the EA peak in the survey spectrum (Figure 1 inset) appears to show s-wave detachment. This apparent s-wave detachment is an artifact of these different peaks collapsing to almost the same radius under the survey conditions, and the high intensity part of the peak still shows an anisotropy value of β = −0.5 ± 0.3.

**Figure 3:**
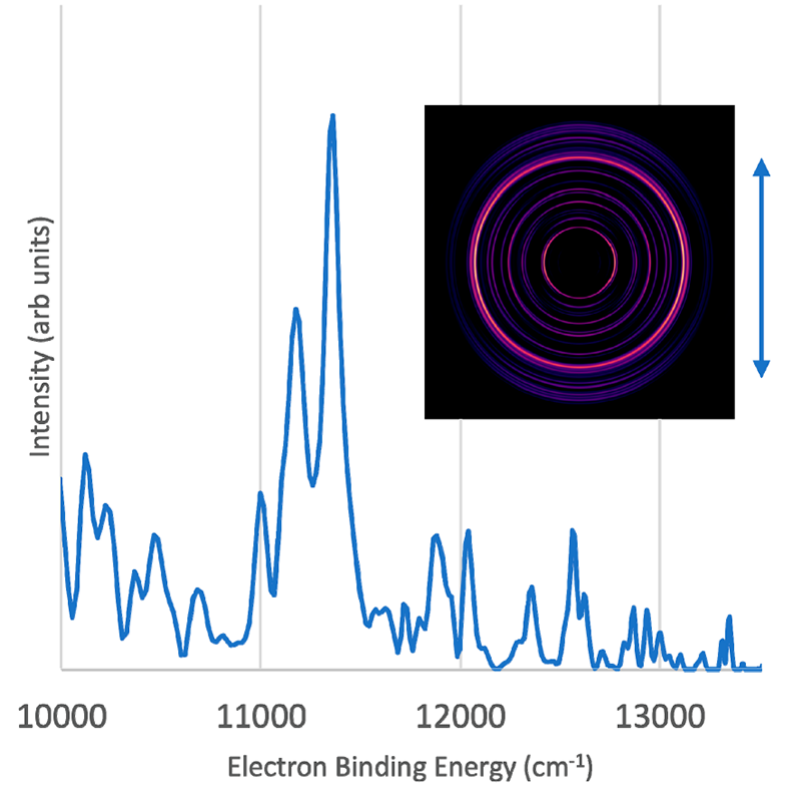
Photoelectron spectrum of Be_3_^–^ taken with a detachment photon energy of 13 605 cm^–1^ (vertical polarization). Inset photo shows the velocity map image produced by MEVELER software

To gain further insight into the experimental data, *ab inito* computations were carried out using the CFOUR package.^47^ Dunning correlation consistent basis sets^48^ were used along with several different coupled-cluster treatments.^45,46,49,50^ Electronic energy predictions are presented in Table 1. Geometry optimizations are presented in Table 2, and the results from vibrational harmonic frequency calculations are included in the Supporting Information (Table S1).

**Table 2:** Bond Lengths of Be_3_ and Be_3_^–^

		bond length (Å)		
method	basis	Be_3_X^1^A_1_′	Be_3_^–^X^2^A_2_″	Be_3_^–^1^2^A_1_′
MRCI	aug-cc-pV5Z	2.203	2.106	2.177
CCSD	aug-cc-pCVTZ	2.218	2.129	2.195
CCSD	aug-cc-pCVQZ	2.201	2.116	2.179
CCSDT	aug-cc-pCVDZ	2.247	2.150	2.216
CCSDT	aug-cc-pCVTZ	2.206	2.114	2.180
CCSDT	aug-cc-pCVQZ	2.191	2.102	2.167

Predictions using the EOMEA-CCSDT method and the aug-cc-pcVTZ basis set were found to provide a better treatment of these transitions than any other method tested when comparing the experimentally determined transition energies to the computational predictions. Interestingly the EOMEA-CCSDT calculations both overestimate the EA of this molecule, with the aug-cc-pcVQZ computation overestimating slightly more than the aug-cc-pcVTZ computation. This may imply that, even with the aug-cc-pcVTZ basis set, the neutral is already almost converged to the CCSDT complete basis limit, while the anion has not reached this limit. Hence, the energy of the anion shifts further down below that of the neutral molecule when the basis set is increased from VTZ to VQZ. More accurate predictions would require a method that accounts for higher order correlation effects like CCSDTQ. As can be seen from the comparison with EOMEA-CCSD, the role of triple excitations is very important to this system, leading to EOMEA-CCSD under-converging the anion states substantially and leading to an EA value approximately 1000 cm^–1^ below the experimental value (not including zero-point energy contributions). It does however appear that EOMEA-CCSD is sufficient to determine the geometry of this molecule, with the bond length being the same within ∼0.02 Å as compared to EOMEA-CCSDT and MRCI. The previously published MRCI/aug-cc-pV5Z results^43^ also agree well with the EOMEA-CCSDT geometries and come close to the experimental values, differing by about 230 cm^–1^ when considering the EA. It is possible that a core–valence basis set would tip the scales in favor of the MRCI method for this molecule, as computations of Be-containing molecules often require the inclusion of core–core and core–valence correlation to be accurately computed, and this phenomenon has been predicted to be substantial for pure Be clusters like Be_3_ as well.

The Be_3_^–^1^2^A_1_′ → Be_3_X^1^A_1_′ and Be_3_^–^X^2^A_2_″ → Be_3_X^1^A_1_′ transitions were observed to have comparable intensities (c.f. Figure 1) under jet cooling conditions, where the X^2^A_2_″ state would be expected to have a greater population than the 1^2^A_1_′ state. Hence, the comparable intensities suggest that the threshold photodetachment cross section for the 1^2^A_1_′Be_3_^–^ → Be_3_X^1^A_1_′ transition is greater than that for Be_3_^–^X^2^A_2_″ → Be_3_X^1^A_1_′. This difference in cross sections is consistent with what may be expected based on the results from computational geometry optimizations.

The relevant bond lengths are shown in Table 2, and the CCSDT (neutral) and EOM-CCSDT (anion) potential energy curves for the symmetric stretch using an aug-cc-pCVTZ basis set are shown in Figure 4. The bond length and shape of the potential curve of the Be_3_^–^1^2^A_1_′ state is nearly identical to the properties of the Be_3_X^1^A_1_′ state, in contrast to the shorter bond length and slightly steeper curvature of the Be_3_^–^X^2^A_2_″ potential energy function. Hence, the near vertical Franck–Condon profile of the Be_3_^–^1^2^A_1_′ → Be_3_X^1^A_1_′ transition may partially account for the higher-than-expected intensity for transitions originating from the anion excited state, as compared to the smaller, less vertical Franck–Condon profile of the Be_3_^–^X^2^A_2_″ → Be_3_X^1^A_1_′ transition. Surprisingly, neither the survey spectrum (Figure 1) nor the higher-resolution spectrum (Figure 2) showed an obvious progression in the totally symmetrical stretch, although an observable progression was predicted by a Franck–Condon simulation based on the symmetric stretch potential energy curves (see the Supporting Information). It is possible that the small peaks at energies above the EA transition were produced by excited vibrational levels in the neutral ground state, but they were not repeatable enough in our study to warrant assignment.

**Figure 4:**
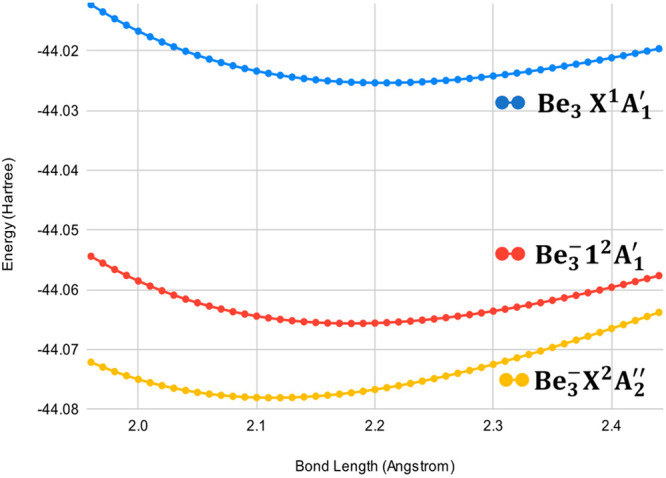
Potential energy curves for the Be_3_^–^ anion and neutral molecule between 1.95 Å and 2.44 Å, computed in 0.01 Å steps along the symmetric stretch. The neutral ground state was computed using CCSDT, and the anion states were computed using EOMEA-CCSDT. All computations were performed in CFOUR using an aug-cc-pCVTZ basis set.

Our ability to consistently image the transition originating from the Be_3_^–^1^2^A_1_′ state suggests that this state has a lifetime of 100 μs or more, based on the time it takes for an anion to reach the position where photodetachment occurs in our apparatus. This is substantially longer than may be expected considering that decay to the anion ground state is (by symmetry) allowed by the electric dipole moment. To determine if this long-lived excited state hypothesis was physically reasonable, the oscillator strength was calculated at the EOMEE-CCSD level with an aug-cc-pCVTZ basis set for the Be_3_^–^1^2^A_1_′ → Be_3_^–^X^2^A_2_″ transition. The oscillator strength was found to be approximately 7.4 × 10^–5^, consistent with an Einstein *A* coefficient of 330 s^–1^ and a radiative lifetime of 3024 μs when using the experimentally determined energy gap, or an Einstein A coefficient of 2320 s^–1^ and lifetime of 431 μs when using the energy gap from the calculation that provided the oscillator strength. In either case, this lifetime is substantially longer than the ∼100 μs flight time in our experiment and longer than the lifetime of any other anion excited state that has a dipole allowed transition to the ground state that we have been able to find in the literature. This long lifetime can be partially explained by looking at the shapes of the 1^2^A_1_′ and X^2^A_2_″ potential energy curves with respect to bond length as seen in Figure 4. The excited 1^2^A_1_′ anion state has substantially different curvature at the bottom of the well as compared to the ground X^2^A_2_″ anion state, which likely leads to a lower vibrational wave function overlap. The inability to relax from the Be_3_^–^1^2^A_1_′ excited state within the time of our experiment, as well as the strong vibrational wave function overlap with the neutral ground state, leads to the Be_3_^–^1^2^A_1_′ → Be_3_X^1^A_1_′ transition showing substantial intensity in all spectra we have taken of this anion.

The photodetachment spectrum and electron velocity map image for Be_4_^–^ are shown in Figure 5. This trace is dominated by a single peak and exhibits a clear preference for the ejection of electrons along the polarization vector of the light used for photodetachment. A higher-resolution trace (included in the Supporting Information, Figure S1) yielded an adiabatic EA of 13 052 ± 50 cm^–1^. The simplicity of this spectrum was readily anticipated by using previously published computational results for Be_4_ and Be_4_^–^. For example, Diaz et al.^19^ examined Be_4_ and Be_4_^–^ using MP2 and ROMP2 methods with basis sets of triple and quadruple-ζ quality. They found that adding an electron to Be_4_ had a minimal effect on the bond length, causing a contraction of just 0.02 Å. Be_4_ has a closed-shell X̃^1^A_1_ ground state and the unpaired electron of Be_4_^–^ resides in an orbital of A_1_ symmetry. The latter is primarily an in-phase linear combination of sp hybrids that point toward the center of the tetrahedron.^19^ This appears to be a nonbonding orbital and yields an X̃^2^A_1_ ground state for the anion. Hence, the dominant single peak of the spectrum and the parallel anisotropy of the Be_4_^–^X̃^2^A_1_→ Be_4_X̃^1^A_1_ photodetachment image are in qualitative agreement with the computational predictions. The quantitative agreement between the measured and calculated EA values for Be_4_ is also acceptable. Diaz et al.^19^ obtained the same value for both the vertical and adiabatic EAs of 13 534 cm^–1^, which is 482 cm^–1^ above the measured value. Our CCSD(T) calculations with the aug-cc-pVTZ basis set gave a vertical EA value of 12 718 cm^–1^, underestimating the measured value by 334 cm^–1^. This result and the calculated harmonic vibrational frequencies for Be_4_ and Be_4_^–^ are listed in Table S2 of the Supporting Information.

**Figure 5:**
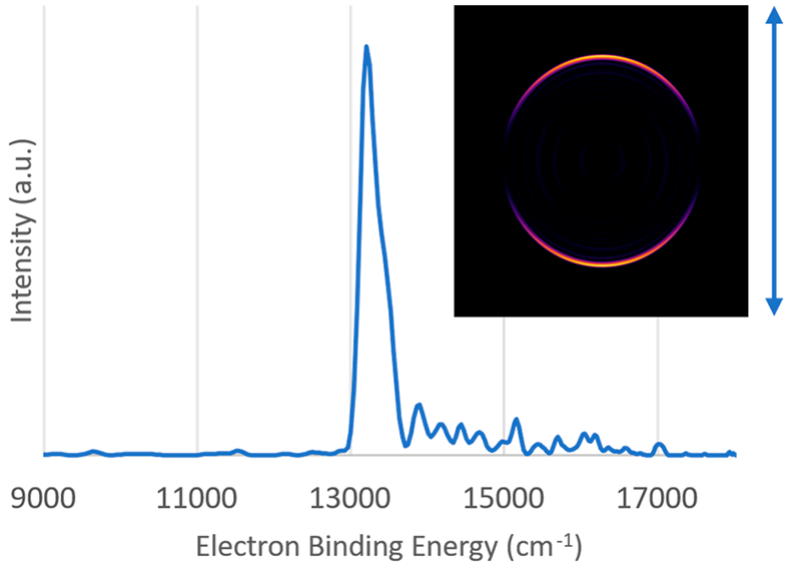
Photoelectron spectrum of Be_4_^–^ taken with a detachment photon energy of 18 182 cm^–1^ (vertical polarization). Inset photo shows the velocity map image produced by MEVELER software.

Higher energy photoelectron spectra for Be_3_^–^ and Be_4_^–^ were recorded by using 355 and 395 nm photodetachment, respectively. These wavelengths provided observation windows for states with internal energies of up to 16 800 cm^–1^ (Be_3_) and 12 264 cm^–1^ (Be_4_). These energies are well above most computational predictions of the triplet excited state energies.^15−17^ In the case of Be_3_, weak transitions were observed in the energy range approximately 6140 to 10640 cm^–1^ above the ground state zero-point level but without strong enough repeatable features to be reliably assigned. We believe this is due to the predicted Jahn–Teller distortion of the Be_3_ geometry in its first excited triplet state (reducing the symmetry to *C*_2*v*_), which lowers the Franck–Condon overlap to these excited states and prevents adequate intensity to assign these features when combined with the already weak signal from Be_3_^–^. In Be_4_, the first excited electronic state has also been predicted to be a triplet state that is subject to Jahn–Teller effects, reducing the symmetry from *T*_*d*_ to either *C*_2*v*_ or *D*_2*d*_ depending on the computational method of prediction. We observed a reasonable amount of spectral activity in the energy range 7950–10 950 cm^–1^ above the Be_4_ origin, in qualitative agreement with expectations of the singlet–triplet gap in the tetramer. However, the spectra were extremely congested, likely due to Jahn–Teller splitting of the triplet.

A number of trends have been delineated in the theoretical studies of small beryllium clusters carried out to date. It has been established that high-level electron correlation treatments are needed for the beryllium dimer and that electron correlation becomes less important as the cluster size increases. The rapid increase of the binding energy in the sequence *N* = 2–4 has been traced to increasing s–p hybridization and many-body interactions. The present experimental results for Be_3_ and Be_4_ help to validate the predictive capabilities of the high-level computational models that revealed the above-mentioned trends. This indicates that our developing qualitative understanding of the bonding in Be_*N*_ clusters is on the right track.

## Experimental Methods

Photodetachment spectra of the Be_3_^–^ and Be_4_^–^ anions were obtained by using a slow electron velocity map imaging spectrometer. This instrument has been described previously.^51,52^ It was recently updated to use an Even-Lavie-type pulsed valve, replacing the previous Jordan-type pulsed valve. Anions were generated by pulsed laser ablation of a pure beryllium rod using the fundamental output of a Nd:YAG laser (1064 nm). The ablation products were entrained in a carrier gas that consisted of approximately 1% nitrogen in argon. The N_2_ was included as it appeared to be an effective third body collider to stabilize cluster anion formation. Anions from the ablation source were expansion-cooled, mass selected, and then photodetached using the harmonics of an Nd:YAG laser (532 and 355 nm) or tunable radiation from a pulsed dye laser (395 nm) or an OPO laser (720–890 nm). The lasers were linearly polarized with a vertical orientation for all measurements. Photoelectron images were accumulated using in-house software that only saved individual “shots” with higher levels of signal to lower the background noise contribution to the image. This was necessary as the yields of beryllium anion clusters were low, and the production was intermittent. The raw velocity map images were processed using the MEVELER^53^ software package, and energy calibrations were performed using the known transitions of S^–^ recorded for various photodetachment wavelengths.

## Supporting Information Available

Calculated vibrational frequencies for Be_3_ and Be_3_^–^, near-threshold photodetachment image and spectrum for Be_4_^–^, calculated Franck–Condon factors for the 2A_1_′–2A_2_″ transition of Be_3_^–^, calculated values for the electron affinity of Be_4_, calculated vibrational frequencies for Be_4_ and Be_4_^–^ (PDF)
Transparent Peer Review report available
