# Supplementary material for: Photoelectron Velocity Map Imaging Spectroscopy of the Beryllium Trimer and Tetramer
Source: J Phys Chem Lett. 2023 Sep 12;14(37):8339–44. doi: 10.1021/acs.jpclett.3c02169 (PMC10518861; doi:10.1021/acs.jpclett.3c02169)
Supplement: Supplementary file 1 — jz3c02169_si_001.pdf [file jz3c02169_si_001.pdf]

Photoelectron Velocity Map Imaging Spectroscopy of the Beryllium Trimer and Tetramer

Noah B. Jaffe*, John F. Stanton† and Michael C. Heaven*

*Department of Chemistry, Emory University, Atlanta, Georgia 30322, USA
†Department of Chemistry - Quantum Theory Project, University of Florida, Gainesville, Florida 32611, USA

Supplemental Information

Table S1. Vibrational Frequencies of Be_3 and Be_3^- (cm^{-1})

Method	Basis	Be_3 X^1A_1'		Be_3^- X^2A_2''		Be_3^- $1^2A_1'$	
		A_1'	E'	A_1'	E'	A_1'	E'
CCSDT	aug-cc-pCVDZ	475	409	568	468	514	394
CCSDT	aug-cc-pCVTZ	512	442	580	486	529	408

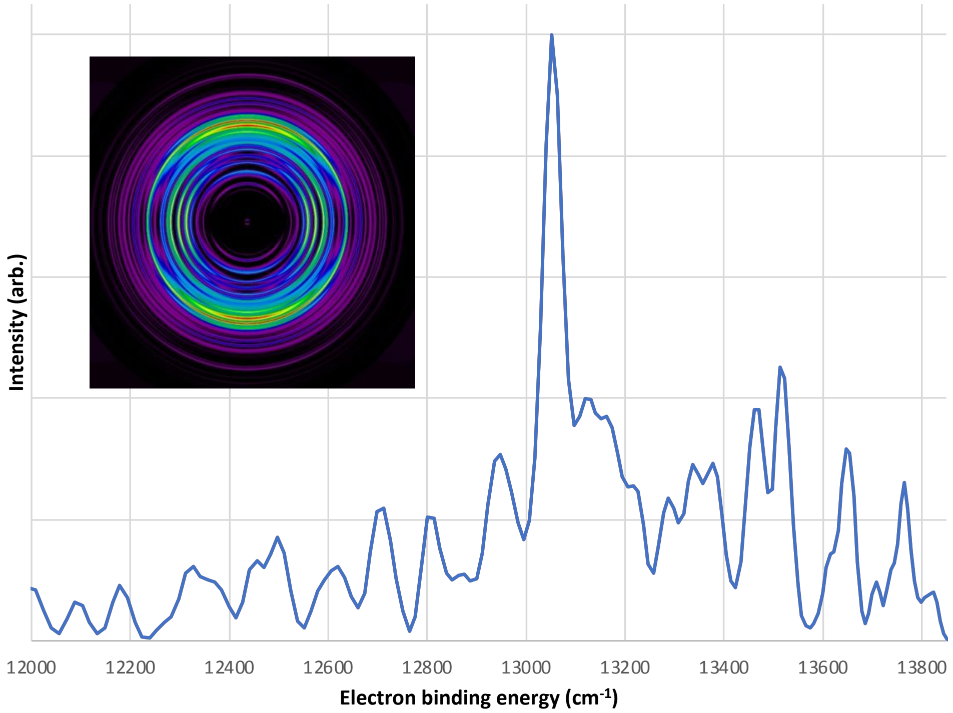

Fig. S1 Photoelectron spectrum of Be_4^- taken with a detachment photon energy of 13800 cm^{-1} (vertical polarization). Inset photo shows the velocity map image produced by MEVELER software.

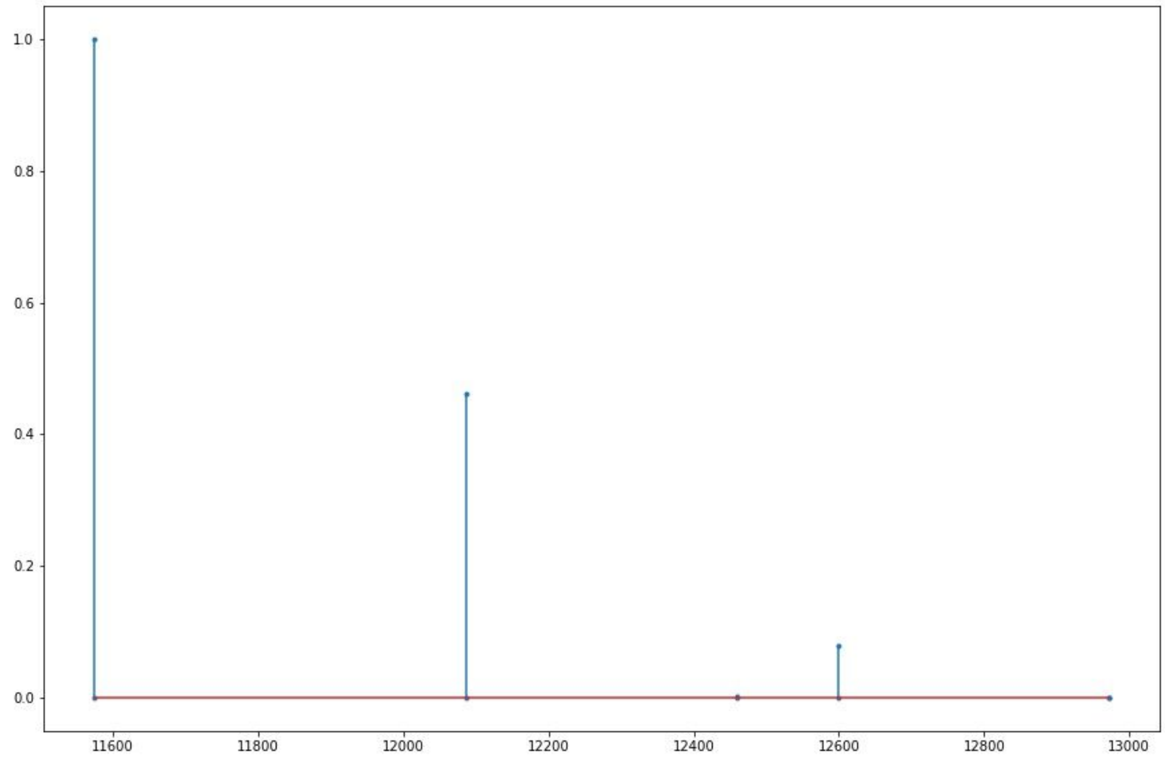

Fig. S2. Relative Franck-Condon factors for the Be_3^- X^2A_2''→Be_3 X^1A_1' photo-detachment transition. Note that the first peak has normalized to 1.0. The symmetric stretch is the only vibrational mode predicted to be active in this spectrum.

Table S2. Calculated values for the electron affinity of Be_4 and the vibrational frequencies for Be_4 and Be_4^-

TRANSITION	METHOD	TRANSITION ENERGY/cm $^{-1}$
$X^2A_1 \rightarrow X^1A_1$ (EA)	CCSD(T)/aug-cc-pVTZ	12718
	MP2/aug-cc-pcVTZ[1]	13611
	ROMP2/aug-cc-pVQZ[1]	13534

Be_4 Ground state Vibrational Frequencies / cm^{-1}

$\boldsymbol{a_1}$	721	CCSD(T)/aug-cc-pVTZ
$\boldsymbol{e}$	456	CCSD(T)/aug-cc-pVTZ
$\boldsymbol{t_2}$	576	CCSD(T)/aug-cc-pVTZ

Be_4^- Ground state Vibrational Frequencies / cm^{-1}

$\boldsymbol{a_1}$	724	CCSD(T)/aug-cc-pVTZ
$\boldsymbol{e}$	428	CCSD(T)/aug-cc-pVTZ
$\boldsymbol{t_2}$	549	CCSD(T)/aug-cc-pVTZ

(1) Diaz, C. C.; Kaplan, I. G.; Roszak, S. Theoretical study of the electron affinities of the alkaline-earth tetramers possessing T_d symmetry: Be_4 and Mg_4. *J. Mol. Model.* **2005**, *11*, 330-334.
