# Supplementary material for: Photoelectron Velocity Map Imaging Spectroscopy of the Beryllium Trimer and Tetramer
Source: J Phys Chem Lett. 2023 Sep 12;14(37):8339–44. doi: 10.1021/acs.jpclett.3c02169 (PMC10518861; doi:10.1021/acs.jpclett.3c02169)
Supplement: Supplementary file 2 — jz3c02169_si_002.pdf [file jz3c02169_si_002.pdf]

jz-2023-02169x.R1

Name: Peer Review Information for "Photoelectron Velocity Map Imaging Spectroscopy of the Beryllium Trimer and Tetramer"

First Round of Reviewer Comments

Reviewer: 1

Comments to the Author

This paper presents the results of velocity map imaging photoelectron spectroscopy of the anions of Be3 and Be4. Although these anions have been the subject of several computational studies, there are no prior experimental characterizations of these anions. For Be3, electron binding energies are determined for the ground and first excited states of the anion, while for Be4, the electron binding energy was determined for the ground state anion. The excited anion state of Be3- is found to be surprisingly long-lived. In addition to the experimental measures the geometries of the ground state neutral and anion states were calculated using the CCSD and CCSDT methods and the electron binding energies were calculated with the EOM-CCSD and EOM-CCSDT. Good agreement is found between the EOM-CCSDT and measured electron binding energies.

Small Be clusters have attracted considerable interest due to large change in the nature of the binding in progressing from the dimer to the tetramer. They have proven important as a testing ground for various electronic structure methods. The availability of accurate experimental values of the electron binding energies of the anions is thus an important addition of our knowledge of these species.

The paper is well written. Publication is recommended in J. Phys. Chem. Lett.

Reviewer: 2

Comments to the Author

This article presents an excellent report detailing a collaborative experimental and theoretical investigation into the structures and excited states of beryllium trimer and tetramer, along with their respective anions. The study showcases cutting-edge experimental techniques and computational calculations, and the authors' interpretation of photoelectron spectra using coupled cluster calculations

is particularly noteworthy. I highly recommend the publication of this manuscript in its current form in JPCL. However, I would suggest that the authors enhance the graphical Table of Contents (TOC) to make it more visually appealing and engaging for JPCL readers. Specifically, the HOMO of Be3- appears rather dull, incorporating captivating photoelectron images obtained in the experiments could greatly improve the appearance of the TOC.

Reviewer: 3

Comments to the Author

The current work carried out a photoelectron imaging study of Be3- and Be4-. Even though this is a hard experiment due to low mass signal, valuable information of several electronic transitions are obtained. The observation of a long life time excited state of Be3- is surprising and is well explained by high level calculation. The joint experimental and theoretical study provide important insight into the electronic structure of beryllium clusters. I recommend acceptance of this paper only with several minor questions and comments:

1.In the survey spectrum(Fig. 1), the origin transition seems to have an isotropic angular distribution (more s partial wave), while in the high resolution spectrum (Fig. 3), this transition becomes more anisotropic (s+d partial wave). Usually, s wave starts to dominate when getting closer to threshold due to the threshold law. Do you have any explanation of this?

2.How do you get the uncertainty? Due to low signal to noise ratio, the peak shape is not well defined. The uncertainty of 30 cm-1 seems small.

3. In the last paragraph of page 8, it states that “For Be3-, Jordan and Simons predicted the lowest energy states were 2A2’’ and separated by 4760 cm-1. ” I am not sure if this is an English problem, but I am a little confused by this sentence. What is the state separated from?

Reviewer: 4

Comments to the Author

This paper took anion photoelectron spectra of Be3- and Be4- in high resolution by slow electron velocity map imaging (SEVI). The assignment of the experimental peaks to the computational results was supported by both electron binding energies and the anisotropy parameters. The obtained electron affinities of both species are in agreement with computational results.

Be3- showed a transition coming from the electronic excited anion to the ground state neutral, in addition to the transition from the ground state anion. The long lifetime and large photodetachment cross section of the excited Be3- were explained by Frank-Condon overlaps using the potential energy curves obtained by calculations.

Reviewer: 5

Comments to the Author

The manuscript “Photoelectron Velocity Map Imaging Spectroscopy of the Beryllium Trimer and Tetramer” by Jaffe et al presents new experimental measurements on the photoelectron distribution of Be3 and Be4 clusters. VMI images were taken for Be3 and Be4 using several wavelengths, enabling SEVI to record high resolution spectra as well as lower energy surveying VMI. The experimental results are supported by high-level theory to describe the dominant electronic transitions that occur.

This manuscript is well written, with a strong introduction that highlights the complexity and challenges of working with Beryllium clusters, as well as the clear need for the measurements taken. Overall, this is impressive experimental/theoretical work and has potential to catch the attention of a diverse group of physical chemists. Thus, this manuscript may be suitable for JPCL, after a few points are addressed:

1. The low energy feature in Be3 anion is attributed to an excited state. I understand that most photoelectron spectra arise from the ground state of the cation into excited states of the neutral. Thus, I believe this is rare, making it a very exciting result. The assignment is supported by the potential energy curves in Figure 4, which lacks a legend or clear description of states. These curves require labels for clarity. Further, the similar shapes between the excited anion and ground state may enhance its FC-cross section, but in similar argument the lack of agreement in curvature between X-anion and X-neutral should create a broadening or asymmetry in the photoelectron peak? Finally, It would be helpful for this energy diagram to also show the lack of a final excited neutral state at this energy from the ground state of the anion with vertical arrows to demonstrate the measured energies.

2. The weaker features of Figure 1 clearly have interesting angular distributions, where B = -2, 0, and 1 seem to appear in the image. Although it is reasonable that they are not assigned, the energies are reported, but not their associated Beta parameters. The Beta parameters should be reported, perhaps in the SI, as they are raw data that is collected in the spectra.

3. Page 5 states that Beta = -0.64 is consistent with the predicted symmetries. This mid-range negative number is not obvious to assign to these symmetries, and should be supported by a reference or description. Similarly, the anisotropy parameter on page 4 (B = 2) is consistent, with an A1-A1 transition. The value of 2, which is an upper limit to B is therefore easier to understand and is justified.

Author's Response to Peer Review Comments:

We thank the reviewers for their careful reading of the paper and helpful feedback. In the following we have summarized the changes made in response to their advice. The relevant comments are reproduced in black text and our responses are given in red.

Reviewers 1 and 4 recommend acceptance of the paper without changes.
Reviewer 2 recommended that the Table of Contents Graphic be improved. We have generated a new image that shows a velocity map image with a superimposed kinetic energy spectrum.

Reviewer: 3

Recommendation: This paper is publishable subject to minor revisions noted. Further review is not needed.

Comments:
The current work carried out a photoelectron imaging study of Be_3^- and Be_4^-. Even though this is a hard experiment due to low mass signal, valuable information of several electronic transitions are obtained. The observation of a long lifetime excited state of Be_3^- is surprising and is well explained by high level calculation. The joint experimental and theoretical study provide important insight into the electronic structure of beryllium clusters. I recommend acceptance of this paper only with several minor questions and comments:

1. In the survey spectrum (Fig. 1), the origin transition seems to have an isotropic angular distribution (more s partial wave), while in the high resolution spectrum (Fig. 3), this transition becomes more anisotropic (s+d partial wave). Usually, s wave starts to dominate when getting closer to threshold due to the threshold law. Do you have any explanation of this?

   This effect is the consequence of overlapping features in the lower resolution survey spectrum. We now comment on this effect on page 5, where the following text has been added-

   "Interestingly, because smaller intensity peaks energetically just below the EA peak have near parallel anisotropy, the anisotropy of the EA peak in the survey spectrum (Fig. 1 inset) appears to show s-wave detachment. This apparent s-wave detachment is an artifact of these different peaks collapsing to almost the same radius under the survey conditions, and the high intensity part of the peak still shows an anisotropy value of $\beta = -0.5 \pm .3$."

2. How do you get the uncertainty? Due to low signal to noise ratio, the peak shape is not well defined. The uncertainty of 30 cm^{-1} seems small.

   We have added the following comment on page 4 to explain how the uncertainties were estimated –

"Multiple images recorded with the same conditions were analyzed and the energies of the assignable features were averaged. The quoted uncertainties are 2σ errors that were determined by converting the uncertainties in pixel space from the raw image to the equivalent error in the electron kinetic energy."

3. In the last paragraph of page 8, it states that "For Be_3^-, Jordan and Simons predicted the lowest energy states were $^2A_2$'' and separated by 4760 cm^{-1}. " I am not sure if this is an English problem, but I am a little confused by this sentence. What is the state separated from?

   A state label was missing. This statement has been deleted in the revised version of the paper.

Reviewer: 5

Recommendation: This paper is probably publishable, but major revision is needed; I do not need to see future revisions.

Comments:
The manuscript "Photoelectron Velocity Map Imaging Spectroscopy of the Beryllium Trimer and Tetramer" by Jaffe et al presents new experimental measurements on the photoelectron distribution of Be3 and Be4 clusters. VMI images were taken for Be3 and Be4 using several wavelengths, enabling SEVI to record high resolution spectra as well as lower energy surveying VMI. The experimental results are supported by high-level theory to describe the dominant electronic transitions that occur.

This manuscript is well written, with a strong introduction that highlights the complexity and challenges of working with Beryllium clusters, as well as the clear need for the measurements taken. Overall, this is impressive experimental/theoretical work and has potential to catch the attention of a diverse group of physical chemists. Thus, this manuscript may be suitable for JPCL, after a few points are addressed:

1. The low energy feature in Be_3 anion is attributed to an excited state. I understand that most photoelectron spectra arise from the ground state of the cation into excited states of the neutral. Thus, I believe this is rare, making it a very exciting result. The assignment is supported by the potential energy curves in Figure 4, which lacks a legend or clear description of states. These curves require labels for clarity. Further, the similar shapes between the excited anion and ground state may enhance its FC-cross section, but in similar argument the lack of agreement in curvature between X-anion and X-neutral should create a broadening or asymmetry in the photoelectron peak? Finally, It would be helpful

for this energy diagram to also show the lack of a final excited neutral state at this energy from the ground state of the anion with vertical arrows to demonstrate the measured energies.

We have added state labels to Figure 4

2. The weaker features of Figure 1 clearly have interesting angular distributions, where β = -2, 0, and 1 seem to appear in the image. Although it is reasonable that they are not assigned, the energies are reported, but not their associated Beta parameters. The Beta parameters should be reported, perhaps in the SI, as they are raw data that is collected in the spectra.

We did not report energies for the weaker features as they were not sufficiently repeatable. Similarly, we do not trust the associated anisotropies.

3. Page 5 states that Beta = -0.64 is consistent with the predicted symmetries. This mid-range negative number is not obvious to assign to these symmetries, and should be supported by a reference or description. Similarly, the anisotropy parameter on page 4 (B = 2) is consistent, with an A1-A1 transition. The value of 2, which is an upper limit to B is therefore easier to understand and is justified.

We have increased the errors for the β parameter and softened the connection to the state symmetries using the following revised text –

"The image also shows a reasonable degree of perpendicular anisotropy, with $\beta = -0.5 \pm .3$, which is supportive of the predicted symmetries of the electronic states involved in this transition."
